# Two firing modes and well-resolved Na^+^, K^+^, and Ca^2+^ currents at the cell-microelectrode junction of spontaneously active rat chromaffin cell on MEAs

**DOI:** 10.1007/s00424-022-02761-0

**Published:** 2022-10-19

**Authors:** Andrea Marcantoni, Giuseppe Chiantia, Giulia Tomagra, Enis Hidisoglu, Claudio Franchino, Valentina Carabelli, Emilio Carbone

**Affiliations:** 1 Department of Drug Science, Laboratory of Cell Physiology and Molecular Neuroscience, N.I.S. Centre, University of Torino, Corso Raffaello 30, 10125 Turin, Italy; 2 Department of Neuroscience, University of Torino, 10125 Turin, Italy

**Keywords:** Extracellular action potentials, Na^+^, Ca^2+^, and K^+^ currents, Ion channel blockers, Cell-electrode interface, AP-clamp recording

## Abstract

We recorded spontaneous extracellular action potentials (eAPs) from rat chromaffin cells (CCs) at 37 °C using microelectrode arrays (MEAs) and compared them with intracellularly recorded APs (iAPs) through conventional patch clamp recordings at 22 °C. We show the existence of two distinct firing modes on MEAs: a ~ 4 Hz irregular continuous firing and a frequent intermittent firing mode where periods of high-intraburst frequency (~ 8 Hz) of ~ 7 s duration are interrupted by silent periods of ~ 12 s. eAPs occurred either as *negative-* or *positive-going* signals depending on the contact between cell and microelectrode: either predominantly controlled by junction-membrane ion channels (*negative-going*) or capacitive/ohmic coupling (*positive-going*). N*egative-going* eAPs were found to represent the trajectory of the Na^+^, Ca^2+^, and K^+^ currents passing through the cell area in tight contact with the microelectrode during an AP (*point-contact junction*). The inward Nav component of eAPs was blocked by TTX in a dose-dependent manner (IC_50_ ~ 10 nM) while the outward component was strongly attenuated by the BK channel blocker paxilline (200 nM) or TEA (5 mM). The SK channel blocker apamin (200 nM) had no effect on eAPs. Inward Nav and Cav currents were well-resolved after block of Kv and BK channels or in cells showing no evident outward K^+^ currents. Unexpectedly, on the same type of cells, we could also resolve inward L-type currents after adding nifedipine (3 μM). In conclusion, MEAs provide a direct way to record different firing modes of rat CCs and to estimate the Na^+^, Ca^2+^, and K^+^ currents that sustain cell firing and spontaneous catecholamines secretion.

## Introduction

Adrenal chromaffin cells (CCs) represent an ideal system for studying the biophysics of ion channels and their role in action potential (AP) generation and neurotransmitter release. Cultured CCs possess sufficiently high densities of Na^+^, Ca^2+^, and K^+^ channels to generate “neuronal-like” APs that regulate catecholamines (CAs) release under electric or secretagogue-induced stimulation [1, 32, 38, 49, 56, 65]. This activity of CCs is at variance with the “neurogenic” regulation of CA secretion ensured by the electrical discharges of the splanchnic nerve, whose cholinergic nerve terminals form contacts with CCs [10]. Acetylcholine (ACh) released by the synaptic contacts depolarizes the CCs through nAChRs activation [25] and triggers AP discharges and CA secretion in amounts proportional to the splanchnic nerve AP frequency [16, 29, 63].

The “non-neurogenic” control is still a matter of dispute, although changes of critical parameters such as extracellular pH, [K^+^], pCO_2_, pO_2_, and circulating or autocrinally released hormones can induce profound functional modifications to CCs firing activity [9, 17, 31, 46, 75]. Cultured CCs and non-innervated adrenal gland slices are preferred to neurogenic preparations for their user-friendly procedures and easy approach to monitor AP firing, membrane ionic currents, and secretory events. This approach allowed to identify the key function of gap-junctions on electrical communications among CCs [15, 31] and highlight the role of a “palette” of ion channels that CCs use to set their resting potential and generate spontaneous or electrically evoked firings [9, 46, 54]. There is now an overall agreement that AP firing in “non-innervated” CCs (cultures and slices) occurs spontaneously following well-resolved tonic and burst-firing modes that drive the release of CAs upon stimulation by various secretagogues [9, 31].

Interestingly, a basal firing similar to that recorded in cultured CCs is evident also in in vivo CCs from intact adrenal glands in anesthetized rats [17]. The extracellular APs (eAPs), recorded with a glass pipette placed on top of the gland in a loose-patch configuration, occur in form of intermittent bursts of variable duration but at variance with isolated CCs, basal firing appears fully driven by splanchnic nerve discharges. Somehow different appear the in vivo eAP recordings using extracellular metal microelectrodes chronically inserted inside the adrenal gland in anesthetized rats [67]. MEA-recorded eAPs undergo continuous basal firing with no evident bursts. Their frequency partially increases following acute adrenocorticotropic hormone addition into blood circulation. Remarkably, the firing of the in situ recorded eAPs changes drastically after the animal undergoes chronic stress tests [67]. In vivo CCs firing exhibits irregular slow bursts of ~ 200 ms duration at a frequency of 0.1–1 Hz that are clearly too slow to be associated with splanchnic nerve discharges expected to occur at 10–15 Hz under stress stimulation. Cautiously, these findings suggest that a non-neurogenic control in in vivo CCs is somehow likely and that microelectrode arrays (MEAs) are an excellent tool to study this CCs activity. MEAs preserve intact the intracellular cell content, provide stable AP recordings of sufficiently high signal-to-noise ratio for days [27, 66], and allow to resolve the different eAPs generated in distinct regions of active neurons (soma, axon, dendrites) [3, 28, 57]. Given the attractive possibility of using MEAs to record stable eAPs in in situ intact adrenal glands in future experiments, a detailed study of eAPs in non-innervated cultured CCs appears a key missing issue that would help solving the relationship between neurogenic and non-neurogenic regulation of CC firings.

Along this line, cultured CCs possess all prerequisites to achieve gold standard eAPs recording using MEAs. Rat CCs have round spherical shape and do not develop neuronal processes even after 4 to 5 days in culture. At variance with neurons, where cell morphology and ion channels distribution varies greatly from somatic, axonal, or dendritic regions, making the interpretation of eAPs quite complex [2, 13, 57], the CCs spherical distribution of ion channels is expected to simplify eAPs analysis. Here we show that rat CCs plated on MEAs at 37 °C generate spontaneous eAPs with frequency comparable to that of intracellularly APs (iAP) recorded with conventional current-clamp recordings at 22 °C. A type of eAP exhibits a negative-going time course in which a quick inward transient component precedes a smaller slow positive wave that ends the eAP. Another type of eAP appears with positive polarity, resembling in shape a scaled iAP. Regardless of the shape and amplitude, eAPs occur into two distinct firing modes: a continuous irregular firing of ~ 4 Hz and a regular intermittent firing of ~ 8 Hz intraburst frequency. In addition, CCs respond to unselective muscarinic agonists with an increased activity, certifying full functionality of the cells on MEAs.

As postulated by Schätzthauer and Fromherz [61] and recent model theories [5, 20, 28, 57], we found that the negative-going eAPs are associated with the inward Nav/Cav and outward Kv/BK currents flowing through the contact area of the cell-microelectrode junction during an eAP. Using selective ion channel blockers, we could resolve the time course of Na^+^, Ca^2+^, and K^+^ currents sustaining AP firings. Despite patch clamp recordings remain the gold standard to obtain detailed information on ion channels contribution to eAPs, our data show that MEA recordings provide direct evidence about the ionic currents sustaining spontaneous eAPs in rat CCs, with the great advantage of collecting simultaneous multiple recordings from populations of cells.

## Materials and methods

### Isolation and culture of rat chromaffin cells on MEAs and plastic dishes

Ethical approval was obtained for all experimental protocols from the University of Torino Animal Care and Use Committee, Torino, Italy. All experiments were conducted in accordance with the National Guide for the Care and Use of Laboratory Animals adopted by the Italian Ministry of Health (Authorization 695/2020-PR). All animals had free access from the shelter to water and food. Every effort was made to minimize animal suffering and the number of animals used. For removal of tissues, animals were deeply euthanized with exposure to a rising concentration of CO_2_ and then rapidly sacrificed by cervical dislocation.

Chromaffin cells were obtained from young (1–3 months) CD female rats and cultured following a modified version of the method by [36]. After removal, the adrenal glands were placed in Ca^2+^ and Mg^2+^ free Locke’s buffer containing (in mM) 154 NaCl, 3.6 KCl, 5.6 NaHCO_3_, 5.6 glucose, and 10 HEPES, pH 7.2, at room temperature. The glands were decapsulated and medullas were precisely separated from the cortical tissue. Medulla digestion was achieved for 70 min at 37 °C in the Locke’s buffer solution containing 0.2 Wünsch units/ml of liberase blendzime (Roche Diagnostics, IN, USA) and 0.2 mg/ml of DNase (Sigma Aldrich, St Louis, MO, USA). The cell suspension was then centrifuged for 5 min at 900 rpm, and washed two times with a Locke’s solution containing 1 mM CaCl_2_ and 10 mg/ml BSA. Cells were then resuspended in 2 ml DMEM supplemented with 15% fetal calf serum (FCS) and plated in four-well plastic dishes or alternatively on MEAs treated with poly-L-ornithine (0.5 mg/ml) and laminin (10 µg/ml in L-15 carbonate) by placing a drop of concentrated cell suspension in the center of each well. After 1 h, 1.8 ml of DMEM supplemented with 15% FCS (Invitrogen, Grand Island, NY, USA), 50 IU/ml penicillin, and 50 µg/ml streptomycin (Invitrogen), 10 μM of Floxuridine and Ara-C hydrochloride (Sigma Aldrich), was added to the wells or MEAs. Cells were then incubated at 37 °C in a water-saturated atmosphere with 5% CO_2_ and used within 2–4 days after plating.

### Voltage-clamp and current-clamp recordings

Voltage-clamp and current-clamp recordings were made in perforated-patch conditions [12] using an Axopatch 200-B amplifier and pClamp 10.0 software programs (Molecular Devices, San Jose, CA, USA). Patch pipettes were made of thin borosilicate glass Kimax 51 (Witz Scientific, Holland, OH, USA) and filled with an internal solution that contained different ions and compounds depending on whether we measured Na^+^, Ca^2+^, or K^+^ currents. For measuring Na^+^ currents in square pulse voltage-clamp experiments, the pipette contained (in mM) 135 CsMeSO_3_, 8 NaCl, 2 MgCl_2_, 20 HEPES, and pH 7.3 with CsOH plus amphotericin B (Sigma Aldrich) and the external bath contained (in mM) 135 TEACl, 2 CaCl_2_, 2 MgCl_2_, 10 glucose, 10 HEPES, 500 μM Cd^2+^, and pH 7.4 with CsOH. For measuring K^+^ currents in square pulse voltage-clamp experiments, the pipette contained (in mM) 135 KAsp, 8 NaCl, 2 MgCl_2_, 20 HEPES, and pH 7.3 with KOH plus amphotericin B and the external bath contained (in mM) 137 NaCl, 4 KCl, 2 CaCl_2_, 1 MgCl_2_, 10 glucose, 10 HEPES, and pH 7.4 with NaOH. For the action potential-clamp (AP-clamp) experiments, the pipette and the bath contained the same solutions used for measuring K^+^ currents. To the external bath were then added 300 nM TTX to block Nav currents and 100 mM TEACl to block Kv and BK currents and record the remaining Ca^2+^ currents. Amphotericin B was dissolved in dimethyl sulfoxide (DMSO) stored at − 20 °C in stock aliquots of 50 mg/ml and used at a final concentration of 500 μg/ml. To facilitate sealing, the pipette was first dipped in a beaker containing the internal solution and then back-filled with the same solution containing amphotericin B. The syringe used for filling the pipettes and containing the internal solution plus amphotericin B was kept cold (0–4 °C) during the experiments.

Pipettes with series resistance of 1–2 MΩ were used to form giga-seals. Recording of currents started when the access resistance decreased below 15 MΩ, which usually happened within 10 min after sealing. Series resistance was compensated by 80% and monitored throughout the experiment. For voltage-clamp experiments, the holding potential (*V*_h_) was − 70 mV throughout the experiments. In the AP-clamp mode, the cell was voltage clamped using one iAP recorded previously during spontaneous firing in current-clamp conditions. This allowed studying the time course of Na^+^, K^+^, and Ca^2+^ currents underlying an AP command [50]. Current traces were filtered using a low-pass Bessel filter set at 1–2 kHz and sampled at 10 kHz. Fast capacitative transients during step depolarization were minimized on-line by the patch clamp analog compensation. Uncompensated capacitive currents were further reduced by subtracting the averaged currents in response to P/4 hyperpolarizing pulses. The indicated voltages were not corrected for the liquid junction potential (LJP), whose estimate is conditioned by the undetermined junction potential of the patch. LJP was 15 mV (absolute value) in current-clamp and voltage-clamp control conditions when measuring AP firing or recording K^+^ and Ca^2+^ and Na^+^ currents. All the experiments were performed at room temperature (22–23 °C).

To determine the type of firing modes (continuous or intermittent) in rat CCs cultured on plastic dishes, current-clamp experiments were performed in perforated-patch clamp conditions using an intracellular solution containing (in mM) 135 KAsp, 8 NaCl, 20 HEPES, 2 MgCl_2_, and 5 EGTA. The external bath contained (in mM) 137 NaCl, 4 KCl, 2 CaCl_2_, 1 MgCl_2_, 10 glucose, 10 HEPES, and pH 7.4 with NaOH. Spontaneous intracellular APs (iAPs) were recorded in current-clamp mode at resting conditions without injecting any current.

### MEA recordings

Multisite extracellular recordings were performed using the MEA system, purchased from Multi-Channel Systems (Reutlingen, Germany). The 60 electrodes array (TiN) is composed by an 8 × 8 square grid with 200 μm inter-electrode spacing and 30 μm electrode diameter [27, 49]. Data acquisition was controlled through MC_Rack software (Multi-Channel Systems Reutlingen, Germany), setting the threshold for spike detection at ± 10 μV and sampling at 10 kHz. Experiments were performed in a non-humidified incubator at 37 °C and 5% CO_2_, without replacing the culture medium (DMEM). Recordings of the spontaneous activity in control conditions were carried out for 5 to 10 min before adding the ion channel blockers or the muscarinic agonist methacholine (100 µM). Recording started after about 1 min from the drug administration.

### Analysis of MEA recordings

The activity of each MEA recording channel (Ch) was visibly analyzed and identified as negative- or positive-going based on the direction of the early eAP component. The eAPs to be analyzed were sorted through the “Threshold Search” routine of Clampfit by setting the level marker 2 to the minimum amplitude to be accepted for analyses. The spike-sorted events were significantly homogeneous in shape and amplitude and were collected in large number of events per channel (from 100 to > 1000 traces) that were subsequently averaged to obtain the final eAP to be analyzed.

Bursts analysis was performed using Neuroexplorer software (Nex Technologies, Littleton, MA, USA) after spike sorting operations. A burst consists of a group of spikes with variable amplitudes, thus we set a threshold of at least 3 spikes and a minimum burst duration of 100 ms. We set interval algorithm specifications such as maximum interval to start burst (0.17 s) and maximum interval to end burst (10 s) recorded in 0.02 s bins.

### Amperometric recordings

Amperometric recordings were performed by using carbon fibers microelectrodes purchased from ALA Scientific Instrument Inc. (Westbury, NY, USA) and a HEKA EPC-10 amplifier (HEKA Elektronik GmbH, Reutlingen, Germany) [7, 30]. Carbon fibers (5 μm diameter) were cut at an angle of 45°, polarized to + 800 mV, and positioned next to the cell membrane. Rat CCs were maintained in standard saline solution, containing (mM) 130 NaCl, 2 MgCl_2_, 10 glucose, 10 HEPES, 2 CaCl_2_, and 4 KCl. For the first 2 min of recordings, the cells were not stimulated and spontaneous exocytic activity was measured. Then the rat CCs were stimulated by using a KCl-enriched solution, containing (mM) 100 NaCl, 2 MgCl_2_, 10 glucose, 10 HEPES, 10 CaCl_2_, and 30 KCl. Amperometric currents were sampled at 4 kHz, low-pass filtered at 1 kHz, and monitored over 120 s. Finally, we analyzed the recordings by using IGOR macros (Wave-Metrics, Lake Oswego, OR, USA) as previously described [8].

### Solutions

External solutions were exchanged as previously reported [8]. Nifedipine, paxilline, TEA-OH, and TEACl were purchased from Sigma Aldrich. Tetrodotoxin (TTX) citrate was purchased from Tocris (Northpoint, Fourth Way Avonmouth, UK) and apamin from Alomone Labs (Jerusalem, Israel).

### Statistics

Data are given as mean ± S.E.M. for *n* numbers of cells. Statistical significance was calculated by using Student’s paired and unpaired *t*-tests (see text and figure legends). Values of *p* ≤ 0.05 were considered significant.

## Results

**Fig. 1:**
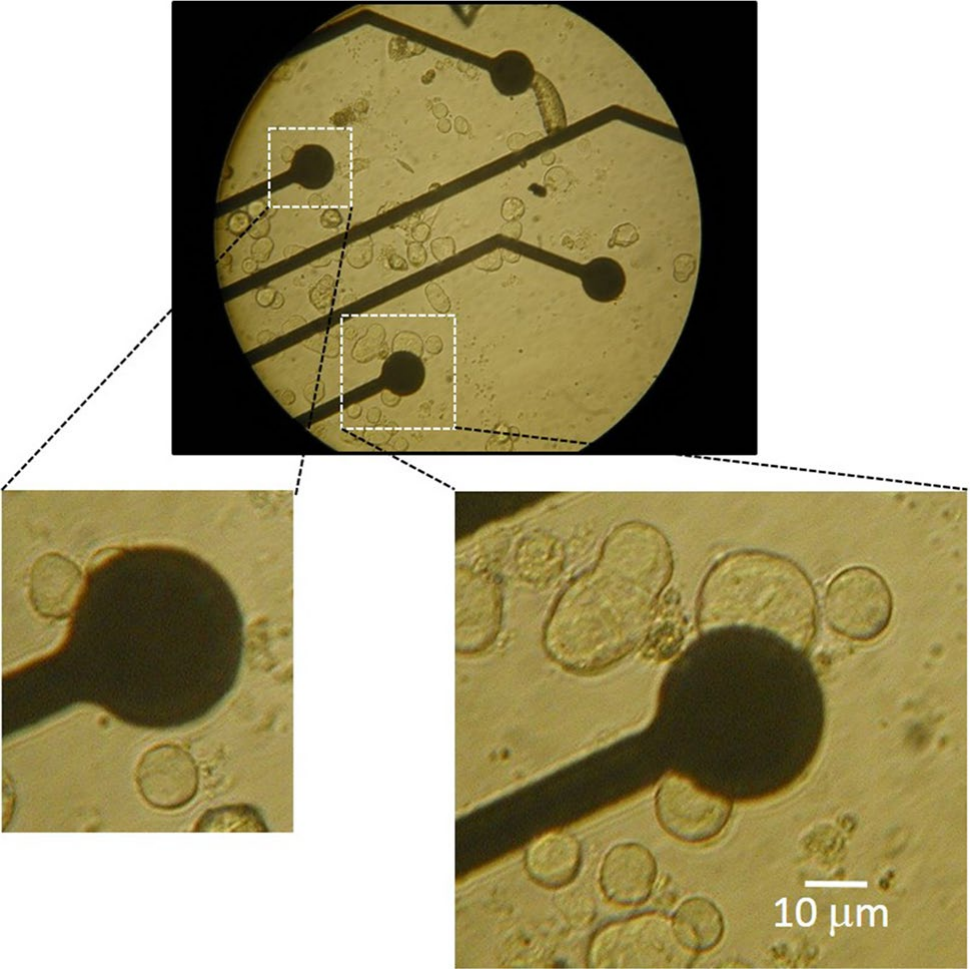
Rat CCs form stable electrical contact on metallic MEAs. Photomicrograph of rat CCs plated on a multielectrode array (MEA1060, MCS, Reutlingen, Germany) for simultaneous recording of eAPs from 60 metallic microelectrodes (diameter: 30 μm). *Insets*: higher magnification of a MEA electrode (black) in contact with two visible chromaffin cells of different size. The cell-microelectrode contact region allows to record spontaneous eAPs of various forms for minutes or hours at 37 °C

Rat CCs preserve their electrical excitability and response to neurotransmitters when cultured for days on metallic TiN MEAs, following the same plating procedure used to maintaining CCs in plastic dishes [7, 8, 49]. Figure 1 shows an example of how CCs are dispersed on the MEA plate and get in contact with the recording microelectrode (MEA channel). Single, pairs, or small group of cells can mechanically contact the low-density MEA electrodes and generate eAPs of different shape and amplitude.

### Spontaneous eAPs of cultured rat CCs on MEAs exhibit different waveforms and modes of firing

**Fig. 2:**
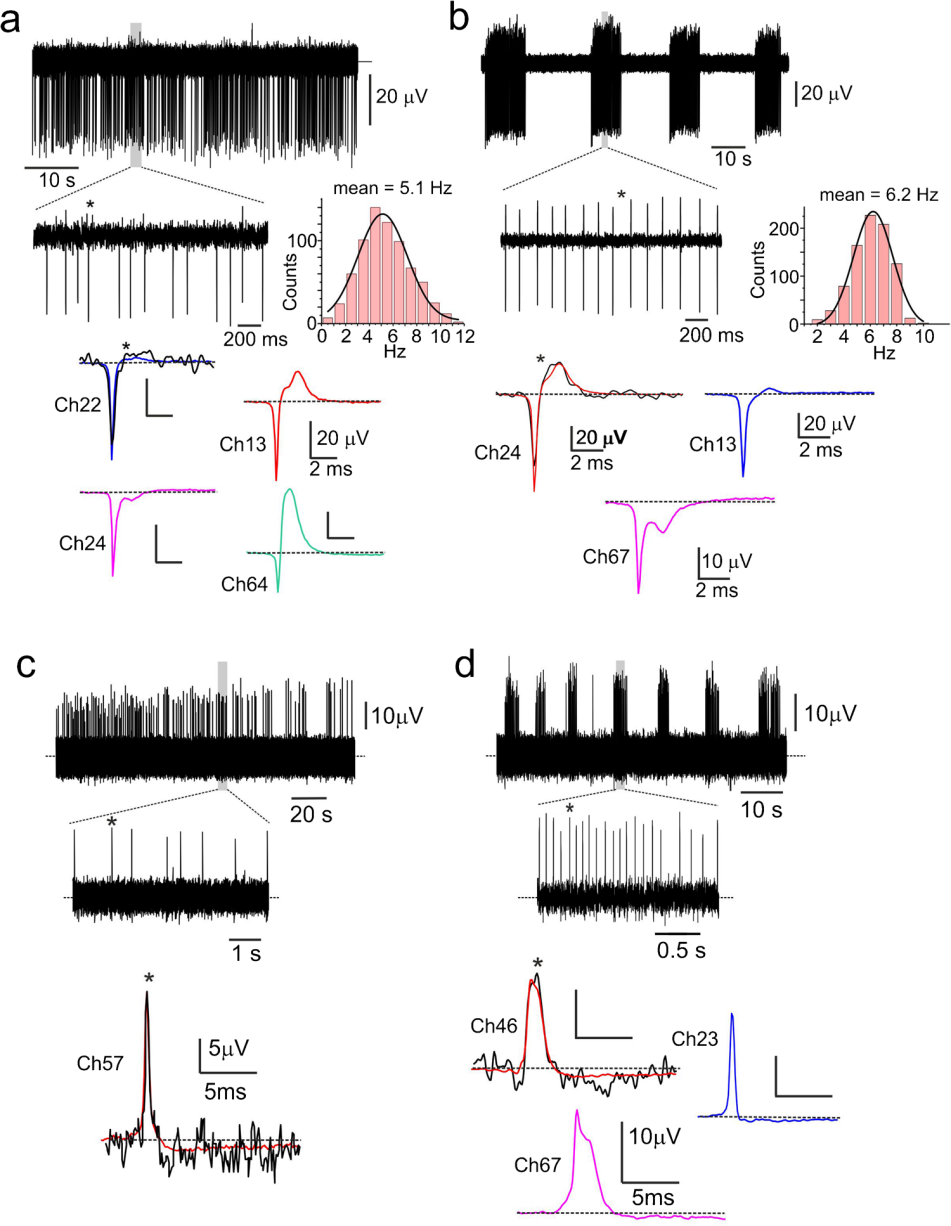
eAPs occurred as negative- or positive-going signals in either continuous or intermittent firing modes. **a** An example of continuous negative-going eAP firing lasting 60 s at two different time scales. In the inset to the bottom, a single eAP (*, noisy trace) overlapped to the average of 158 eAP traces recorded by the same electrode (Ch22, blue trace) on an expanded time scale. To the right, the firing frequency distribution best fit with a Gaussian function (mean = 5.1 Hz; σ = 2.1 Hz). The shape of eAPs was extremely variable as shown by the averaged eAPs in the inset: Ch24 magenta (171 traces), Ch13 red (246 traces), and Ch64 green (132 traces). **b** An example of intermittent negative-going eAP firing lasting 100 s at two different time scales. To the right, the intraburst firing frequency distribution best fit with a Gaussian function (mean = 6.2 Hz; σ = 1.5 Hz). In the inset to the bottom, a single eAP (*, noisy trace) overlapped to the average of 317 eAP traces recorded by the same electrode (Ch24, red) on an expanded time scale. As for the continuous firing mode, the shape of averaged eAPs was extremely variable: Ch67 magenta (171 traces) and Ch13 blue (236 traces). **c**, **d** Two examples of positive-going eAP firing, displaying continuous (**c**) or intermittent (**d**) firing mode lasting 170 s and 75 s, respectively. In panel **c** (below), on an expanded time scale, is shown the single (*, noisy trace) overlapped to the averaged eAP of 142 traces (red) of the top continuous positive-going eAPs. In panel **d** (below) is shown a single eAP (*, noisy trace) and the average of 161 eAP traces recorded from the same electrode (Ch46, red trace). As for the negative-going eAPs, the shape of averaged traces was extremely variable: Ch67 magenta (222 traces) and Ch23 blue (190 traces)

**Fig. 3:**
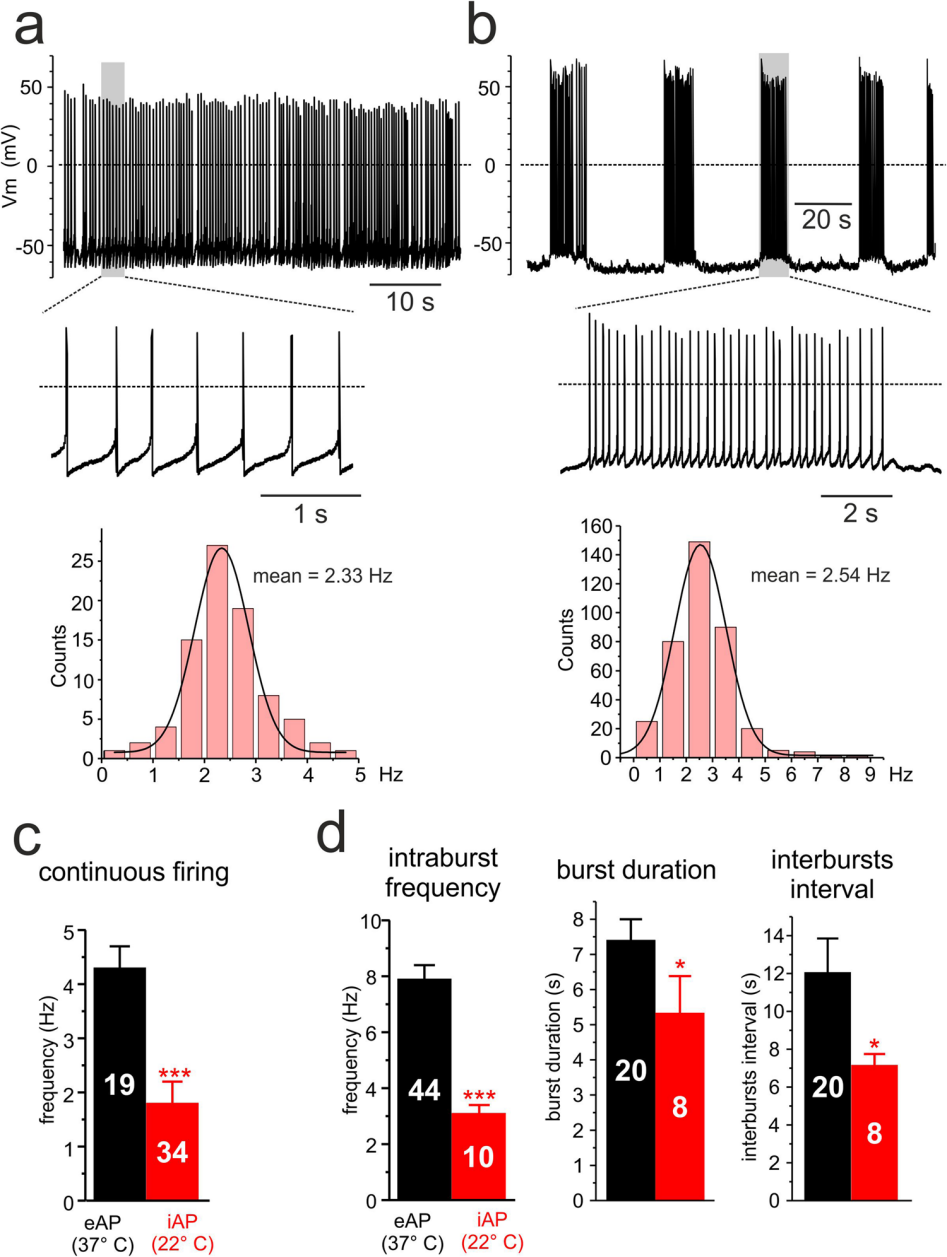
Two distinct firing modes recorded in perforated-patch conditions from spontaneously firing rat CCs. **a** An example of continuous iAP firing at two different time scales to resolve the shape of the single iAP. To the bottom, the firing frequency distribution best fit with a Gaussian function (mean = 2.33 Hz; σ = 0.53 Hz). **b** An example of intermittent iAPs firing at two different time scales. To the bottom, the intraburst firing frequency distribution best fit with a Gaussian function (mean = 2.54 Hz; σ = 0.96 Hz). **c** Comparison of the mean frequencies of continuous eAPs firing (negative- and positive-going) recorded on MEAs at 37 °C (black bar) and continuous iAP firing recorded in perforated patches at 22 °C (red bar; ***p < 0.001, unpaired Student’s *t*-test). **d** Comparison of the mean intraburst frequency, burst duration, and interbursts interval characterizing the intermittent firing. Data are derived from negative- and positive-going eAPs recorded on MEAs at 37 °C (black bars) and iAPs recorded on perforated patches at 22 °C (red bars) (*p < 0.05, ***p < 0.001, unpaired Student’s *t*-test)

Spontaneous eAP firings were evident while monitoring randomly for minutes or hours the activity of the 60 active channels (Ch) of a MEA plate. At variance from the intracellularly APs (iAPs) recorded with glass pipettes [49], eAPs occurred in two opposite waveforms: either as dominant downward (negative-going eAP; Fig. 2a,b) or as dominant upward deflections (positive-going eAP; Fig. 2c,d) with two distinct modes of firing for both eAP waveforms. A firing pattern exhibited the typical “slow irregular” continuous firing observed in iAPs (Fig. 3a) (see also [1, 32, 49]). The eAPs frequency during continuous firing was normally distributed (inset in Fig. 2a) with mean frequency of 4.3 ± 0.4 Hz (*n* = 20) at 37 °C, which is a factor 2.4 greater than the mean frequency estimated on spontaneous iAPs recorded with glass pipette at 22 °C (1.8 ± 0.4 Hz, *n* = 34) (Fig. 3c; *p < 0.05). This firing mode occurred more frequently (61% over 479 cells; Fig. 2a,c) with respect to the second type of firing observed in the rest of active MEA channels (39%). This latter was characterized by an intermittent firing in which eAPs appear in high frequency bursts of 7.4 ± 0.6 s mean duration, followed by long quiescent periods of 12.1 ± 0.6 s (*n* = 20; Fig. 2b,d). The eAPs frequency during the bursting period was regular and normally distributed (inset in Fig. 2b), with mean frequency of 7.9 ± 0.5 Hz (*n* = 44) regardless of the direction of eAPs (positive- or negative-going; Fig. 2b,d). Notice that mean frequencies of negative- and positive-going eAPs in the continuous and intermittent firing mode were not statistically different. Interestingly, the intermittent firing was less often observed in iAPs recorded from cultured rat CCs on plastic dishes (~ 5% of cells; Fig. 3b) (see [32, 49]). iAPs had mean intraburst frequency of 3.1 ± 0.3 Hz (*n* = 10) and lasted 5.3 ± 1.1 s with interburst intervals of 7.1 ± 0.6 s (*n* = 8) (Fig. 3d). As for the continuous firing, the frequency of the intermittent firing on MEAs was 2.5-fold greater than that of iAPs due to the higher temperature of MEA recordings (37° vs. 22 °C; Fig. 3d).

### The negative-going eAPs

Despite positive- and negative-going eAPs provide the same information concerning the all-or-none nature of APs (frequency of the events), we focused our attention on the analysis of the negative-going eAPs for two reasons: they occurred more frequently in both the continuous and intermittent firing modes (57% of cells) and their waveform was easier to interpret. As shown in Fig. 2a and b (colored traces), negative-going eAPs were characterized by an early inward component of 40–100 µV amplitude, lasting less than 1 ms, followed by a delayed outward component of 10–20 µV and 2–5 ms duration. The negative-going inward component varied greatly in amplitude but preserved the narrow transient time course in all the analyzed recordings. The outward component appeared also of different amplitude but had variable kinetics. Representative negative-going eAPs are shown in the inset of Fig. 2a and b with positive components of large (> 20 μV; red traces) and small amplitude (< 10 μV; blue), with no positive component (magenta) or a positive component larger than the negative peak (green).

Following the common interpretation of MEA-recorded eAPs from neurons [28, 57, 61], the negative-going eAP represents the trajectory of the inward Na^+^/Ca^2+^ and outward K^+^ currents passing through the membrane portion of the cell in contact with the microelectrodes when the intracellular membrane potential undergoes an all-or-none AP. The variable forms and amplitudes of the inward/outward components depend on the size of CC area in contact with the microelectrode and on the density of Na^+^, Ca^2+^, and K^+^ channels expressed at the contact region (cell-electrode junction). As indicated by P. Fromherz in leech neurons [23, 61], these eAPs are generated by a “C-type” neuron-electrode junction where high densities of Nav and Kv channels are expressed and contribute to the total current passing through the neuron-electrode junction. Ad hoc neuron-electrode junction models nicely mimic several eAP waveforms by simply changing the Na^+^/K^+^ conductance ratio [53, 61]. More precisely, the eAP recorded by MEAs reflects the voltage drop that is generated by a current source (the firing chromaffin cell) in contact with the MEA [20, 28, 57].

It is interesting to notice that the negative-going eAPs in CCs do not exhibit the fast initial capacitive component (fast positive transient) that anticipates the inward Na^+^/Ca^2+^ current often recorded in central neurons (see Fig. 2a,b in [23]). The lack of early capacitive currents in rat CCs is most likely due to the small cell capacitance of round-shaped CCs (4–12 pF) compared with mature neurons (50–200 pF) and to the slow rising of the AP trajectory generated by the open subthreshold Cav1 channels that anticipates the upstroke of iAPs [49, 50, 72].

### The positive-going eAPs

Less straightforward is the interpretation of the positive-going eAPs characterized by an early positive upward deflection followed by a small negative component. This type of eAP was recorded in 43% of CCs (*n* = 479). In some case, the eAP resembled the trajectory of iAPs of variable width and amplitude (see examples in panels 2c,d). In other cases, the positive-going eAPs appeared as pure biphasic capacitive transients. In general, these eAPs resemble the MEA recordings generated by capacitive (A-type) or ohmic coupling (B-type) in leech neurons [53, 61]. In the case of CCs, the positive-going eAPs are likely generated by active cells with significant ohmic coupling at the cell-microelectrode junction. Indeed, in rat CCs, we rarely observed biphasic eAPs with an early brief positive peak (0.5 to 1 ms) followed by a negative transient of about the same short duration that are typically recorded in neurons and attributed to pure capacitive coupling with no ionic currents passing through the cell membrane at the junction (see Fig. 5 in [61]). It is also important to mention that as shown in Fig. 1, more than one cell can couple to the same microelectrode and may thus give rise to complex eAPs. For instance, negative and positive eAPs may overlap. We found that this occurs in 11% of the total MEA recordings (*n* = 479). In 75% of cases, they occurred as negative continuous eAPs overlapped to positive continuous or intermittent eAPs while 15% occurred as negative and positive intermittent eAPs. The remaining 10% appeared in all the other possible combinations.

### Rat CCs spontaneously secrete catecholamines under basal conditions

**Fig. 4:**
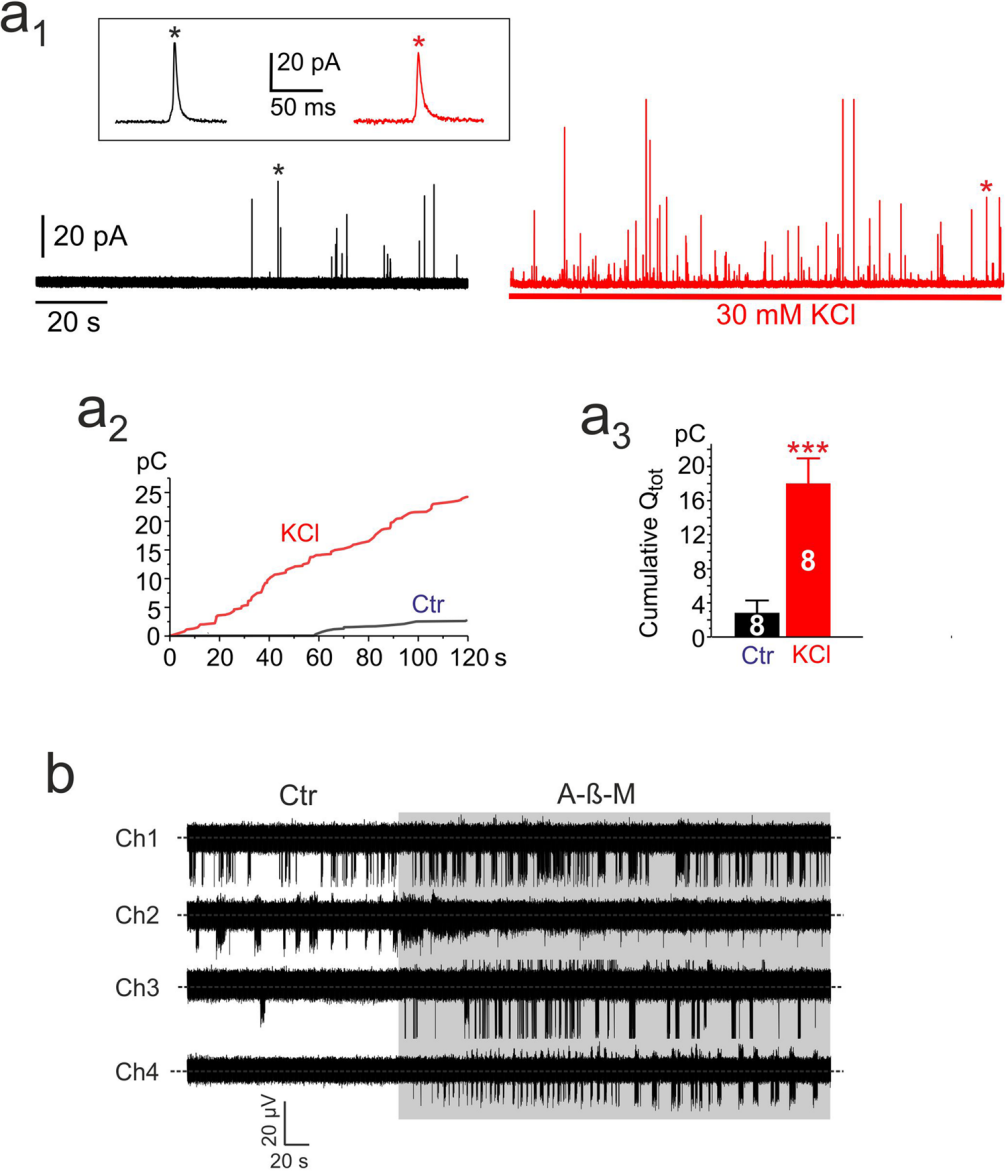
Rat CCs secrete catecholamines and respond to muscarinic agonists on MEAs. **a** RCCs preserve their spontaneous catecholamine (CA) secretory activity, recorded with a carbon fiber microelectrode (CFE) directly positioned on top of a CC plated on plastic dishes. **a**_**1**_ CA release in form of amperometric spikes is evident during resting conditions and increases markedly during addition of 30 mM KCl. *Inset on top*: single amperometric spikes recorded under basal (black; *) or KCl stimulation (red; *). The characteristic kinetic parameters of spikes did not change significantly during KCl stimulation (see text). **a**_**2**_ Overlap of cumulative secretion plots obtained from the amperometric recording of panel **a**_**1**_ during control (black line) and KCl application (red line). **a**_**3**_ Mean total cumulative charges calculated at the end of 120 s recording (Q_tot_) in control (black bar) and KCl application (red bar) (*n* = 8 cells; ***p < 0.001, paired Student’s *t*-test). **b** Rat CCs eAP firing activity of four representative MEA electrodes (Ch; channel) recorded in control conditions (white window) are compared with those recorded in the presence of methacholine (A-ß-M, 100 µM) (grey window). Notice the variable effects from cell to cell

After having shown that rat CCs undergo spontaneous electrical activity on MEAs (Fig. 2), we next tested whether CCs possess a basal exocytotic activity and how much this activity increases by adding 30 mM KCl, using carbon fiber microelectrodes (CFEs) to detect CAs release. A total of 30 mM KCl steadily depolarized rat CCs [8] and abolished iAP firing in few seconds [44]. CCs were maintained in the same standard saline solution used for eAP recordings on MEAs with 2 mM [Ca^2+^]_o_ (see “Materials and methods”). Under these conditions, by positioning the CFE on top of the CCs plated on plastic dishes, we could detect amperometric spikes associated with the basal spontaneous exocytotic events for minutes in 43% of cells (13 out of 30 cells). The frequency of spontaneous spikes was fivefold lower of that induced by cell depolarization with 30 mM KCl (0.09 ± 0.04 vs. 0.48 ± 0.08 Hz, respectively; *n* = 13, *p < 0.05, paired Student’s *t*-test; Fig. 4a1), while the size and kinetics of single amperometric spikes were not significantly altered. Maximal current was 26.5 ± 1.3 pA for KCl stimulated cells and 28.2 ± 3.1 pA for spontaneous activity (not shown). The total charge (Q) of a single spike was respectively 0.22 ± 0.01 pC vs. 0.21 ± 0.02 pC and the slope of the rising phase (m) was 13.7 ± 1.3 nA/s during spontaneous release vs. 11.6 ± 0.6 nA/s during KCl stimulation. The full width at half maximum (*t*_1/2_) was 6.6 ± 0.5 ms and 6.9 ± 0.2 ms, and time to attain the current peak (*t*_p_) was 3.4 ± 0.2 ms vs. 3.8 ± 0.1, respectively.

We also determined the time course of cumulative secretion (Fig. 4a2) and calculated the mean total cumulative charges released after 120 s recording (*n* = 8) and found a sixfold increase of the total charges (*Q*_tot_) in 30 mM KCl with respect to basal secretion (Fig. 4a3). These results prove that the size, the granules content, and the time course of exocytotic spikes do not vary during spontaneous or KCl-evoked release and that under physiological conditions (2 mM [Ca^2+^]_o_), rat CCs undergo spontaneous CA release. Interestingly, the amount of cells that fire spontaneously in an intermittent mode (39%) matches closely the percentage of cells that secrete CAs (43%) even in the absence of KCl stimulation, pointing out the importance of intermittent AP firing in regulating CA secretion. This proves that under physiological conditions, rat CCs possess an intrinsic autorhythmic activity and basal secretory activity due to the proper expression of pace-making and AP-generating ion channels [46].

### Rat CCs on MEAs are sensitive to the muscarinic agonist methacholine: eAPs activity is potentiated in firing cells and recruited in silent cells

Considering the relevance of muscarinic receptors in the modulation of CCs firing frequency [9, 58], we next tested whether rat CCs on MEAs preserve their functional property to respond to muscarine by changing their spontaneous firings (Fig. 4b). As shown in Fig. 4b, the addition of the unspecific muscarinic agonist methacholine (A-β-M, 100 μM) causes three different typologies of responses: a sustained potentiation of spontaneous firing (Ch1), a reduction of firing (Ch2), and a recruitment of firing from apparently silent or weakly active CCs (Ch3 and Ch4) that correspond to the diverse responses to muscarine (30 μM) of current-clamped mouse CCs (I. Mendez-Lopez, A. Garcia, E. Carbone; unpublished observations). Assuming that the number of active electrodes in the presence of A-ß-M represents the total cells potentially able to generate eAPs, on *n* = 10 MEA plates, we estimated that 68 ± 8% of the electrodes were already active before A-ß-M application, and with the agonist, the percentage of active MEAs further increased to ~ 100%. We can therefore expect that among all the CCs present in the medulla, 68% of them are spontaneously active and an additional 32% can be recruited to generate eAPs if stimulated with muscarine.

### TTX-sensitive Nav channels are responsible for the early negative component of eAPs

**Fig. 5:**
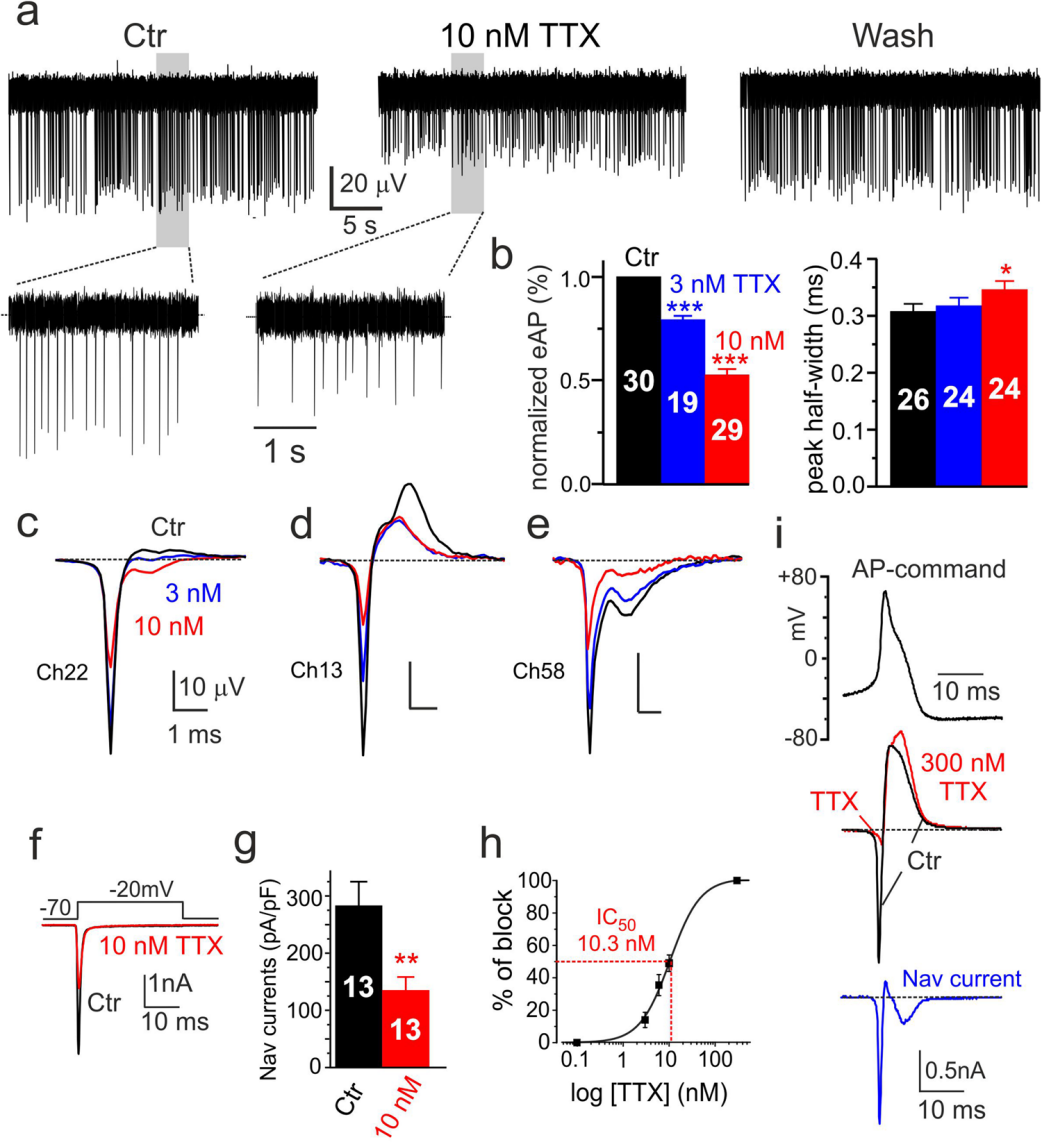
Increasing concentrations of TTX cause a progressive block of the negative eAP peak. **a** The blocking effects of 10 nM TTX on the negative-going continuous eAP firings of a rat CC at different time scales. Notice the partial reduction of the negative eAP amplitude and firing frequency and nice recovery after wash. **b** Percentage reduction of the normalized negative eAP component and peak half-width at 3 and 10 nM TTX. Numbers in the bars indicate the number of cells analyzed for each experiment (*p < 0.05; ***p < 0.001, paired Student’s *t*-test). **c**–**e** Examples of TTX blocking effects on eAPs of different shape: the control eAPs are averages of 157 (Ch22), 135 (Ch13), and 225 traces (Ch58). In CCs expressing no visible outward K^+^ currents (Ch58), the peak reduction is on both negative peaks, suggesting that the eAP is mainly carried by Nav channels (see text). **f** Nav currents in control and during addition of 10 nM TTX recorded in voltage-clamp conditions at − 20 mV. **g** Percentage of Nav current block by 10 nM TTX (**p < 0.01, paired Student’s *t*-test). **h** Dose dependence of Nav currents block by TTX. The continuous curve is a best fit dose–response with IC_50_ = 10.3 nM. **i** The time course of Nav currents (blue trace; bottom) recorded during an AP command (black; top) in voltage-clamp conditions. Nav currents are obtained by subtracting from control currents (black; middle) the currents persisting after adding 300 nM TTX to fully block Nav channels (red; middle). Notice the “dual negative peak” of the Nav current associated with the rising and falling phase of the AP (see text)

Rat and mouse CCs express Nav1.3 and Nav1.7 TTX-sensitive sodium channels [46, 52, 71]. Both channels are fully blocked by 300 nM TTX [47, 52, 73] and AP firing is blocked in the majority of cells at these high TTX concentrations. However, although markedly attenuated, a firing driven by Ca channels (Ca-spikes) still could persist in a fraction of rat [1] and mouse CCs [70, 71]. To assay the contribution of Nav channels to the eAP waveforms, we tested the effects of lower doses of TTX (3 to 10 nM) in order to preserve the spontaneous firing while inducing robust block of Nav1.3/Nav1.7 channels. Figure 5a shows the blocking effects of 10 nM TTX on eAPs when applied to a continuously firing rat CC. TTX reduced reversibly by ~ 50% the amplitude of the early negative peak of eAPs (Fig. 5b) causing also a partial reduction of the amplitude of the positive component (Fig. 5c,d) and a decrease of firing frequency (Fig. 5a, grey windows). On average, 3 nM (blue traces) and 10 nM TTX (red) blocked by 21 ± 2% (*n* = 19) and 47 ± 3% (*n* = 29) (***p < 0.001) the negative peak amplitude, respectively (Fig. 5b), regardless of the cell firing mode (continuous or intermittent). The block of the negative peak was accompanied by a slight broadening of the half-width. On average, this latter increased by 13%, from 0.31 ms in control to 0.35 ms with 10 nM TTX (*p < 0.05) (Fig. 5b, right), most likely due to the smaller amplitude and nearly double broadening of spontaneous iAPs induced by 10 nM TTX as observed in mouse CCs (see Fig. 7D in Vandael et al., 2015).

Similar percentages of block occurred to Nav currents recorded in voltage-clamp conditions using square-pulse commands (Fig. 5f,g). TTX blocked Nav currents with an IC_50_ = 10.3 nM (Fig. 5h), which is comparable to the IC_50_ of Nav current block in mouse CCs (see Fig. 2A in [71]). This suggests that the early negative wave of the eAP represents the voltage drop associated with the TTX-sensitive inward Nav currents flowing through the cell-electrode point contact [61, 62]. Figures 5d and 5e show the effects of 3 and 10 nM TTX on two cells in which, in one case, there was a dual positive peak component with the large peak strongly attenuated by 10 nM TTX (panel d). In the second case, there were two negative-going waves of different size, both attenuated by the toxin. The reduction of the positive wave in Fig. 5d is most likely the consequence of the delayed rise and decreased amplitude of APs induced by the block of Nav channels that attenuates K^+^ channels activation. In panel 5e, the control eAP shows two negative waves (black trace) that are both depressed by TTX (blue and red), suggesting strong contribution of Nav channels to the early and late negative waves and a not well-resolved outward K^+^ current component.

### The negative-going eAP reflects the time course of Nav currents flowing through the cell-electrode junction during spontaneous APs

The eAP of Fig. 5e is impressively similar to the Nav currents measured intracellularly in rat CCs under voltage-clamp conditions using an AP command (top trace in Fig. 5i). Under “AP-clamp” conditions, Nav currents can be quantified by subtracting from control (black trace, Fig. 5i-middle) the TTX-resistant current persisting after full block of Nav currents with 300 nM TTX (red). The resulting trace is the time course of Nav currents (blue, Fig. 5i-bottom). The first transient negative wave derives from the progressive increase of Nav channel open probability (*P*_o_) times the negative driving force for Na^+^ (*V*-*E*_Na_), which progressively decreases during the rise of the AP. The Nav inward current reaches its negative peak when *P*_o_ obtains its maximum (*P*_o_ = 1 around 0 mV) and starts declining following the decrease of *V*-*E*_Na_, while *P*_o_ remains equal 1. Once reached the zero baseline at *V* = *E*_Na_, the current turns outward to reach a maximum at the peak of AP, which in the case of Fig. 5i is slightly larger than *E*_Na_ and *V*-*E*_Na_ is somewhat positive. Following that, the Na^+^ current returns inward during the repolarization phase of the AP due to the progressive increase of *V*-*E*_Na_. Nav currents attain a second negative peak and finally return to the baseline because of the progressive lowering of *P*_o_ below 0 mV. The striking similarity between the eAP waveform of Fig. 5e and the time course of Nav currents recorded in the AP-clamp mode strengthens the idea that the negative-going eAPs reflect the voltage drop caused by the ionic currents passing through the membrane at the cell-electrode contact region during spontaneous APs.

As shown in Fig. 5a, 10 nM TTX caused a clear reduction of the frequency. In cells exhibiting continuous firing, the firing frequency decreased from 5.1 ± 0.02 to 3.9 ± 0.02 Hz (*n* = 8; *p < 0.05, paired Student’s *t*-test), while in cells with the intermittent firing, the intraburst frequency decreased even more markedly, from 7.7 ± 0.05 to 4.8 ± 0.07 Hz (*n* = 8; *p < 0.05, paired Student’s *t*-test). The mean frequencies were estimated pooling together negative- and positive-going eAPs since the inhibitory effect of TTX on frequency was independent of the direction of eAPs. In other rat CCs, the toxin could induce stronger decreases or even full block of eAPs with usually partial recovery of both the frequency and amplitude. Concerning the two positive eAP components associated with K^+^ currents, TTX reduced the amplitude of either one or both positive waves in *n* = 25 cells, most likely due to the delayed rise and smaller amplitude of APs with TTX.

### The Kv and BK currents sustaining spontaneous iAPs

**Fig. 6:**
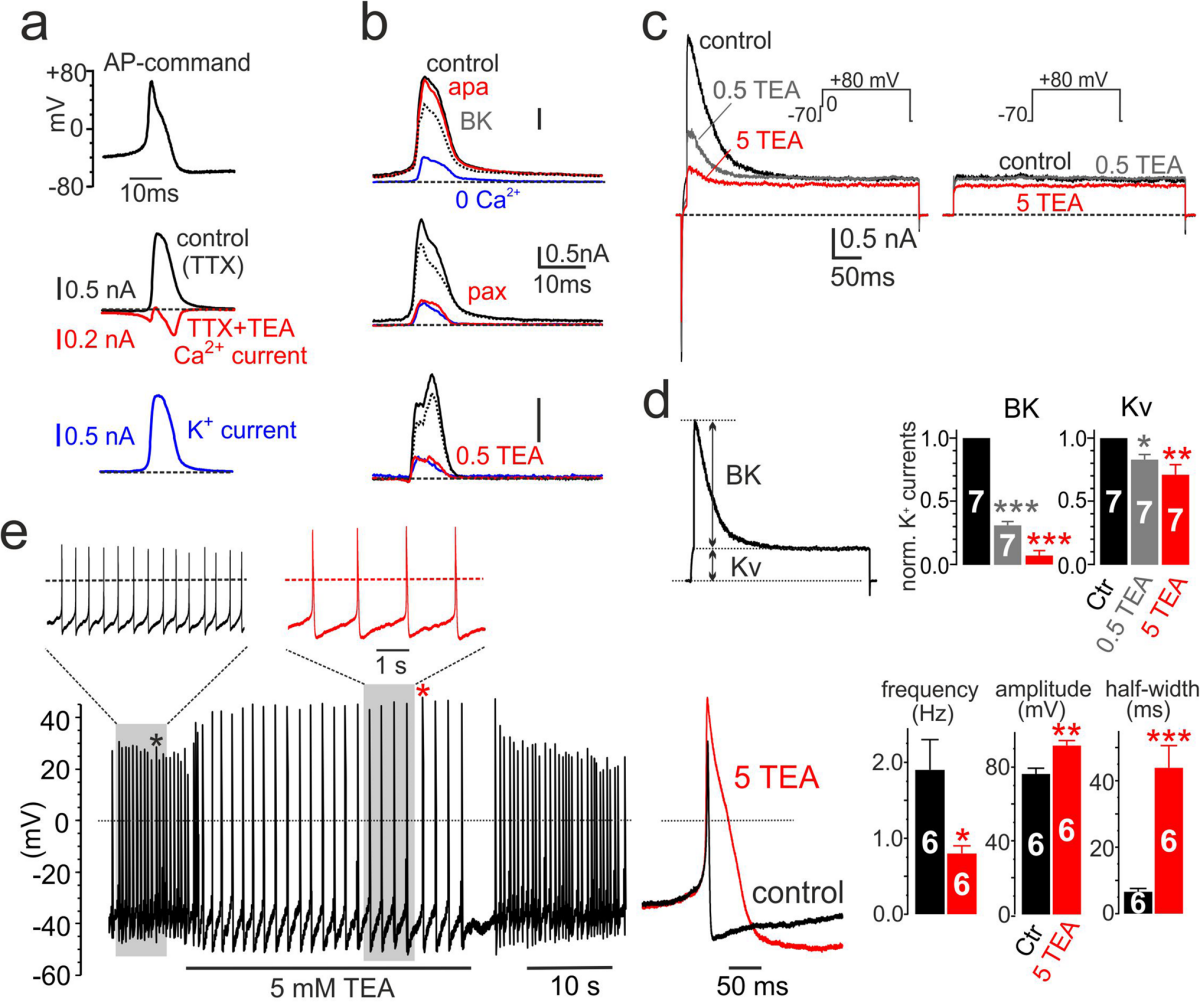
Separation of Kv, BK, and Cav currents using whole cell AP-clamp recordings and the inhibitory effects of 5 mM TEA on iAP firing. **a** Whole-cell voltage-clamp experiments to separate inward Ca^2+^ currents from outward K^+^ currents during an AP command (top black trace). In the middle are shown the control currents recorded in the presence of 300 nM TTX (black trace) and the inward Ca^2+^ currents recorded after adding 100 mM TEA to the solution containing TTX (red). To the bottom are shown the outward K^+^ currents (blue) obtained by subtracting control (TTX) from Ca^2+^ currents (TTX + TEA). **b** Voltage-clamp experiments to separate Kv from BK currents during an AP command by either adding solutions containing 0 Ca^2+^ (top), 100 nM paxilline (middle), or 0.5 mM TEA (bottom). *Top*: control currents (black trace) with 100 nM apamin (red) or 0 Ca^2+^ solution (blue). Subtraction of the current in 0 Ca^2+^ from control uncovers the outward BK currents (grey dashed trace). *Middle*: same protocol using 100 nM paxilline in place of 0 Ca^2+^. Notice the potent block by paxilline that is comparable to that of 0 Ca^2+^. *Bottom*: same protocol as above using 0.5 mM TEA in place of paxilline or 0 Ca^2+^ to block selectively BK currents. Notice in all three cases the dominant contribution of BK current (grey dashed) to the total outward K^+^ current. **c** Voltage-clamp experiments using classical double pulse protocols to determine the amplitude of transient BK and steady-state Kv currents in control, 0.5 and 5 mM TEA. To the left are shown the overlapped BK and Kv currents recorded at + 80 mV after a 10 ms Ca^2+^ preloading step to 0 mV (see inset). To the right are the overlapped Kv currents activated from a potential step to + 80 mV. **d** Estimate of BK and Kv current amplitude separated as indicated to the left. Notice the strong reduction of BK currents vs. the Kv currents in 0.5 (grey bars) and 5 mM TEA (red): *p < 0.05, **p < 0.01, ***p < 0.001, paired Student’s *t*-test. **e** Current-clamp recordings of spontaneous iAPs in control and during addition of 5 mM TEA. TEA decreased the firing frequency, increased the amplitude, and the half-width of iAPs as shown on the bar graphs: *p < 0.05, **p < 0.01, ***p < 0.001, paired Student’s *t*-test

Concerning the K^+^ currents (Kv, BK, SK) contributing to the spontaneous eAPs, we first tested the effects of various K^+^ channel blockers (TEA, apamin, paxilline) on the K^+^ currents recorded during iAP commands in whole-cell perforated-patch conditions (AP-clamp; top panel of Fig. 6a). Outward currents measured in the presence of TTX (black trace, middle) were fully blocked by adding 100 mM TEACl. The remaining inward current (red trace) was the expected inward Ca^2+^ current sustaining the iAP, which exhibited a prominent negative peak during the decaying phase of the iAP (see Marcantoni et al., 2010). The net outward K^+^ current obtained by subtracting the TEA-resistant currents (red trace) from control (black) is shown at the bottom of panel a (blue). K^+^ current attains a peak value soon after the fast AP repolarization (+ 40 mV) and last until the hyperpolarization phase is complete. On average, the total K^+^ outward currents had amplitudes of 1.4 ± 0.2 nA (*n* = 17) and half-width durations of 8 ± 0.8 ms (*n* = 17).

To separate voltage-gated Kv currents from Ca^2+^-dependent BK and SK currents [49, 55] and quantify the contribution of BK and SK channels, we tested the effects of paxilline, low doses of TEA (0.5 to 5 mM), and apamin (200 nM) [49, 64, 72]. In parallel with the K^+^ channel blockers, we also tested the effects of Ca^2+^ free (0 Ca^2+^) Tyrode’s solution (Ca^2+^ replaced with Mg^2+^) to estimate the contribution of Kv currents persisting in 0 Ca^2+^ and the Ca^2+^-dependent K^+^ currents that are non-active in the absence of external Ca^2+^. Apamin (200 nM) had nearly no effect on the K^+^ currents sustaining the AP (red trace; Fig. 6b-top) while zero Ca^2+^ markedly reduced the total K^+^ current (blue). Peak K^+^ currents in 0 Ca^2+^ decreased by about 80% regardless of the K^+^ channel blocker tested (Fig. 6b) (from 1.4 ± 0.2 nA in control to 0.3 ± 0.1 nA in 0 Ca^2+^; *n* = 17, **p < 0.01, paired Student’s *t*-test). Taken together, these data suggest that SK channels do not contribute to the K^+^ currents sustaining the iAP while Kv channels contribute only partially (20%). As confirmed by the strong effects of the selective BK channel inhibitor paxilline (1 μM) and 0.5 mM TEA (Fig. 6b-middle/bottom), K^+^ outward currents are mainly controlled by BK channels in rat CCs (grey dashed traces in Fig. 6b).

The use of 0.5 mM TEA to selectively block BK channels was suggested by the results of the voltage-clamp experiments shown in Fig. 6c. Increasing doses of TEA (0.5, 1, and 5 mM) block progressively the transient BK current at + 80 mV activated by the 20 ms prepulse depolarization to 0 mV (Fig. 6c-left) but preserves the Kv current activated at + 80 without prepulse (Fig. 6c-right). Separation of BK from Kv currents (Fig. 6d-left) confirms that 0.5 mM TEA blocks ~ 70% of BK and 16% of Kv channels (Fig. 6c; grey bars in Fig. 6d-right), while 5 mM TEA blocks potently BK channels (93%) and only partially Kv channels (~ 30%, red bars in Fig. 6d-right). Thus, 5 mM appears an optimal TEA concentration to effectively block BK currents by preserving enough Kv currents to sustain the AP firing repolarization, which is central for running spontaneous eAP recordings on MEAs. To confirm this, we tested the effects of 5 mM TEA on iAPs and found that the firing is indeed preserved. iAPs were higher and broader and occurred at lower frequency (Fig. 6e-left). A total of 5 mM TEA reduced the firing frequency by nearly half (from 1.9 ± 0.4 to 0.8 ± 0.1 Hz; *n* = 6; *p < 0.05) but increased the iAP amplitude from 76.3 ± 3.1 to 91.7 ± 2.7 mV (*n* = 6; **p < 0.01) and the half-width from 6.6 ± 1.0 to 43.9 ± 6.7 ms (*n* = 6; ***p < 0.001) (Fig. 6e-right).

### BK channels mainly contribute to the positive eAP component associated with cell repolarization

**Fig. 7:**
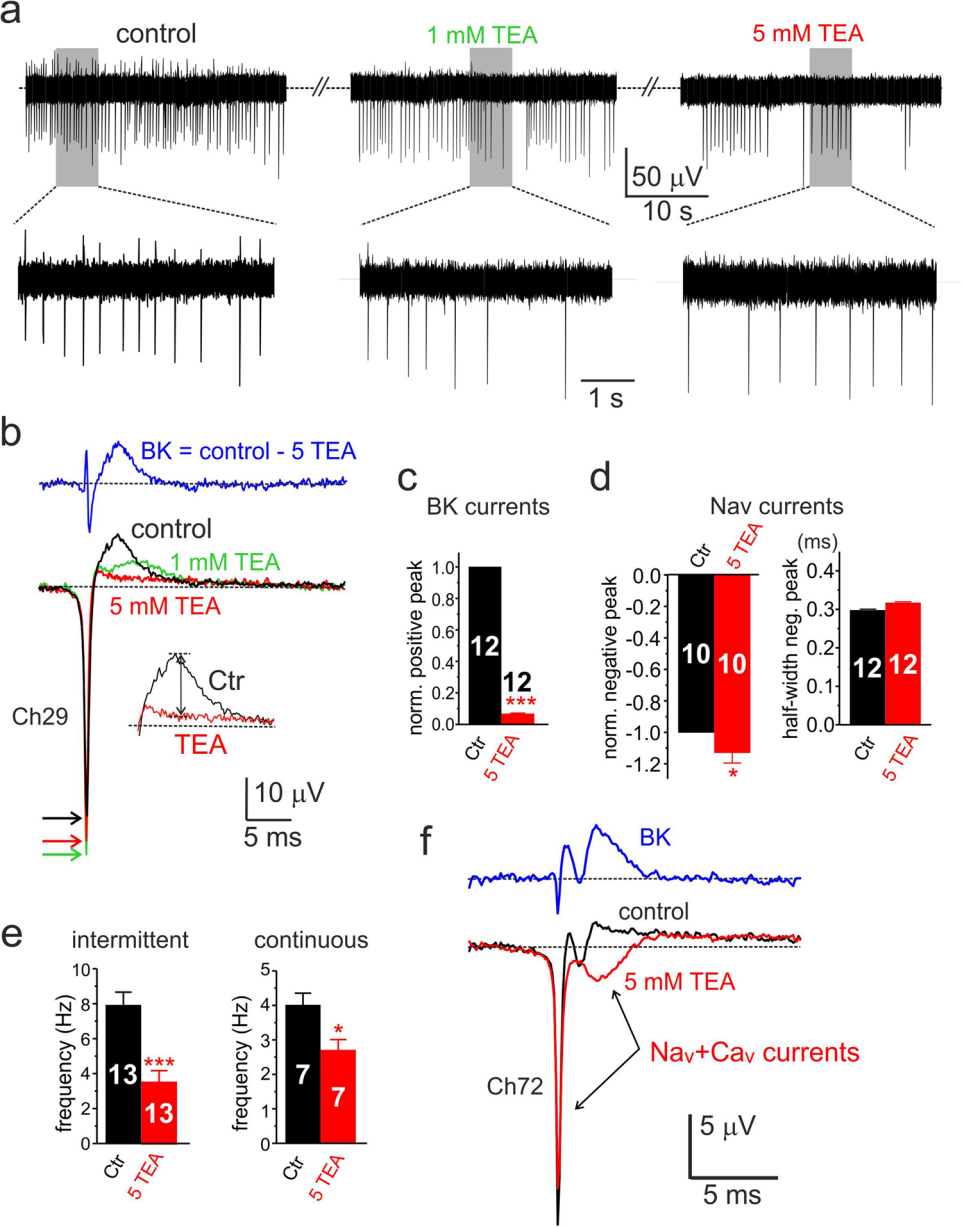
Five mM TEA reduces the positive peak and the firing frequency of eAPs. **a** Effects of 1 and 5 mM TEA on the negative-going continuous eAP firings of a rat CC at different time scales. One and 5 mM TEA cause a progressive decrease of the late positive eAP component, a decrease of firing frequency, and a small increase of the early negative eAP component. **b** Averages of eAPs recorded in control (black; 152 traces), 1 mM (green; 118 traces), and 5 mM TEA (red; 94 traces). On top, the BK current-associated eAP obtained by subtracting control from 5 TEA eAPs. Colored arrows indicate the peak of the negative eAP component. The inset shows how was estimated the amplitude of the positive component associated with BK currents. **c** Mean values of the normalized positive peak eAP estimated as indicated in the inset of panel **b** (***p < 0.001, paired Student’s *t*-test). **d** Mean values of the normalized negative peak and half-width of eAPs (*p < 0.05, paired Student’s *t*-test). **e** Mean frequencies of intermittent (intraburst) and continuous firing (*p < 0.05, ***p < 0.001, paired Student’s *t*-test). **f** Average eAPs recorded from a cell possessing a small positive eAP component. Addition of 5 mM TEA uncovers an eAP with two negative peaks typical of Nav and Cav currents in iAP-clamp recordings. The displayed eAPs are averages of 256 (control; black) and 221 traces (5 mM TEA; red). On top is shown the difference between control and 5 mM TEA associated with the BK component

We next tested how the block of BK channels alters the shape and firing frequency of eAPs. Figures 7a,b show an example of how increasing doses of TEA (1 to 5 mM) cause an increased block of the outward eAP component and reduced firing frequency. The block with 5 mM TEA (see inset in Fig. 7b) was almost complete (93 ± 0.01%, *n* = 12, ***p < 0.001) (Fig. 7c), regardless of the firing mode.

As expected from the block of outward K^+^ currents, 5 mM TEA partially increased the peak amplitude of the inward component by nearly 13% (arrows in panel b) with no effect on the half-width duration (Fig. 7d). As for the iAPs, 5 mM TEA reduced the frequency of eAPs in both the continuous and intermittent mode (Fig. 7e). The mean intraburst frequency of intermittent firings decreased markedly, from 8.0 ± 0.7 to 3.6 ± 0.6 Hz (*n* = 13; ***p < 0.001), while the frequency of continuously firing decreased from 4.0 ± 0.4 to 2.7 ± 0.3 Hz (*n* = 7; *p < 0.05). Given that 5 mM TEA was able to block most of the outward eAP component, in some CCs expressing low densities of K^+^ channels, addition of TEA could uncover a second inward eAP peak (Fig. 7f), likely associated to the Nav and Cav currents passing during the falling phase of iAP (Fig. 5e). The red trace in Fig. 7f is likely associated to the Nav, Cav, and Kv channels spared by 5 mM TEA. In conclusion, the action of 5 mM TEA on eAP reflects closely the waveform changes detected on the inward and outward currents recorded in rat CCs under AP-clamp.

**Fig. 8:**
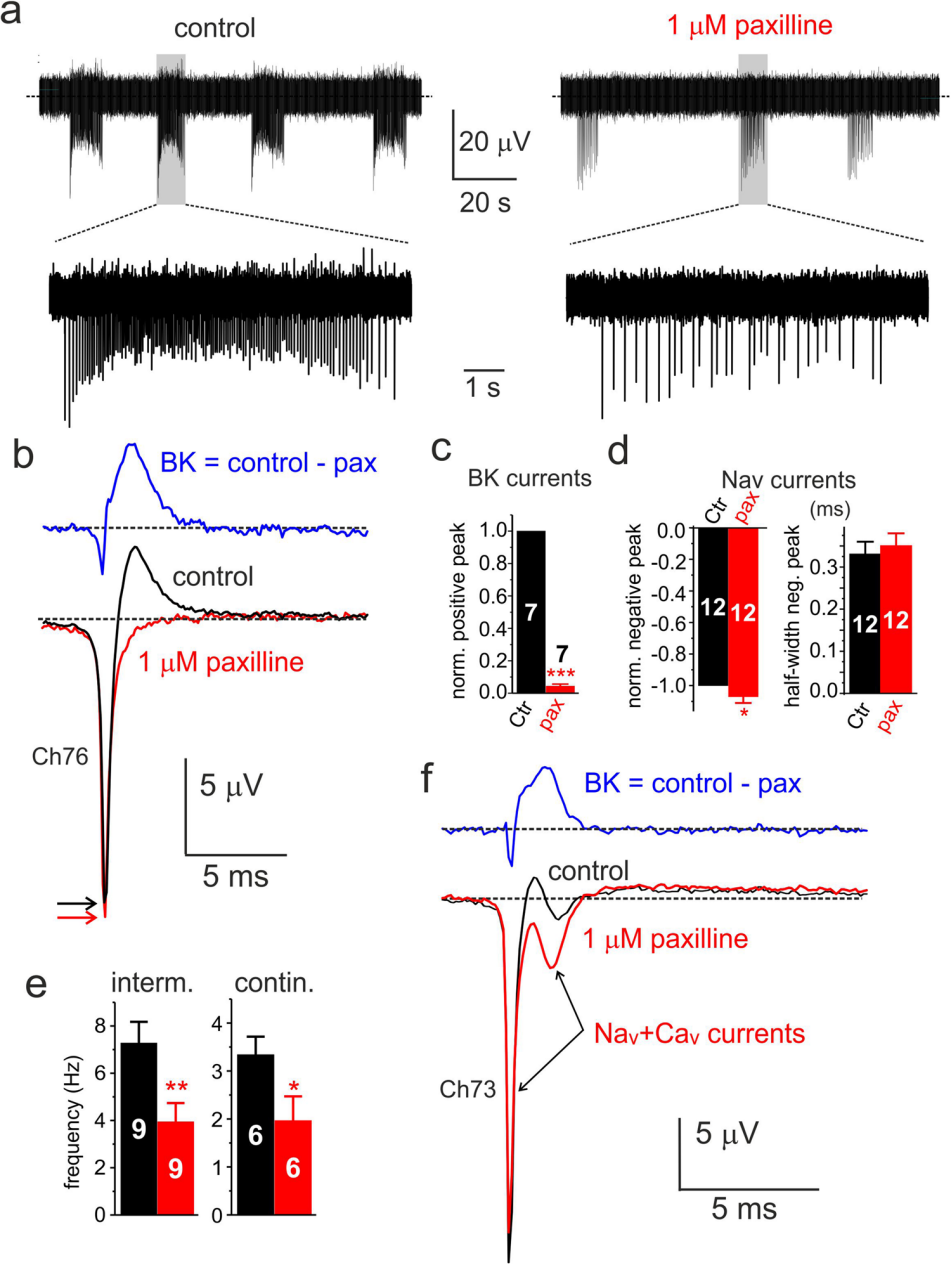
Paxilline acts on eAPs like low doses of TEA. **a** Effects of 1 μM paxilline on the negative-going continuous eAP firings of a rat CC at different time scales. Paxilline produces the same effects of 5 mM TEA: a marked decrease of the late positive eAP component, a decrease of firing frequency, and a small increase of the early negative eAP component. **b** Averages of eAPs recorded in control (black; 505 traces) and 1 μM paxilline (red; 214 traces). On top, the BK current-associated eAP obtained by subtracting the eAPs of control from 1 μM paxilline. Colored arrows indicate the peak of the negative eAP component. **c** Mean values of the normalized positive peak eAP estimated as indicated in the inset of panel 7b (***p < 0.001, paired Student’s *t*-test). **d** Mean values of the normalized negative peak and half-width of eAPs (*p < 0.05, paired Student’s *t*-test). **e** Mean frequencies of intermittent (intraburst) and continuous firing (*p < 0.05, **p < 0.01, paired Student’s *t*-test). **f** Average eAPs recorded from a cell with a small positive eAP component. Addition of 1 μM paxilline uncovers eAPs with “two negative peaks” typical of Nav and Cav currents recorded in iAP-clamp. The displayed eAPs are averages of 254 traces (control; black) and 438 traces (paxilline; red). On top is shown the difference between control and 1 μM paxilline associated to the BK component

To confirm that the outward eAP component is mainly associated with BK channels, we also tested the effects of paxilline (0.2 to 1 μM). Paxilline selectively blocks BK channels in mouse [50] and rat CCs [49] by favoring the closed-channel conformation [76] and is routinely used to quantify BK currents in a broad variety of cells [26, 43]. Paxilline markedly blocked the outward component of negative-going eAPs by 96 ± 0.1% (*n* = 7; ***p < 0.001, Fig. 8b,c) and increased the early negative peak of eAP carried by Nav and Cav channels by ~ 7% (Fig. 8b,d) with no detectable effects on the half-width (Fig. 8d). Paxilline reduced also by ~ 50% the mean intraburst frequency of intermittent firings and the mean frequency of continuous firings (Fig. 8e). Interestingly, in cells in which the outward BK component was less prominent (Fig. 8f), the block of BK channels could uncover a second negative-going eAP of smaller amplitude (red trace in Fig. 8f). This eAP, with two negative-going components, is most likely associated with the Nav and Cav currents passing through the cell-microelectrode interface during the rising and falling phase of iAP and is similar to the eAPs of Fig. 5e,5i, and 7f.

### SK channels do not contribute to the positive eAP component

**Fig. 9:**
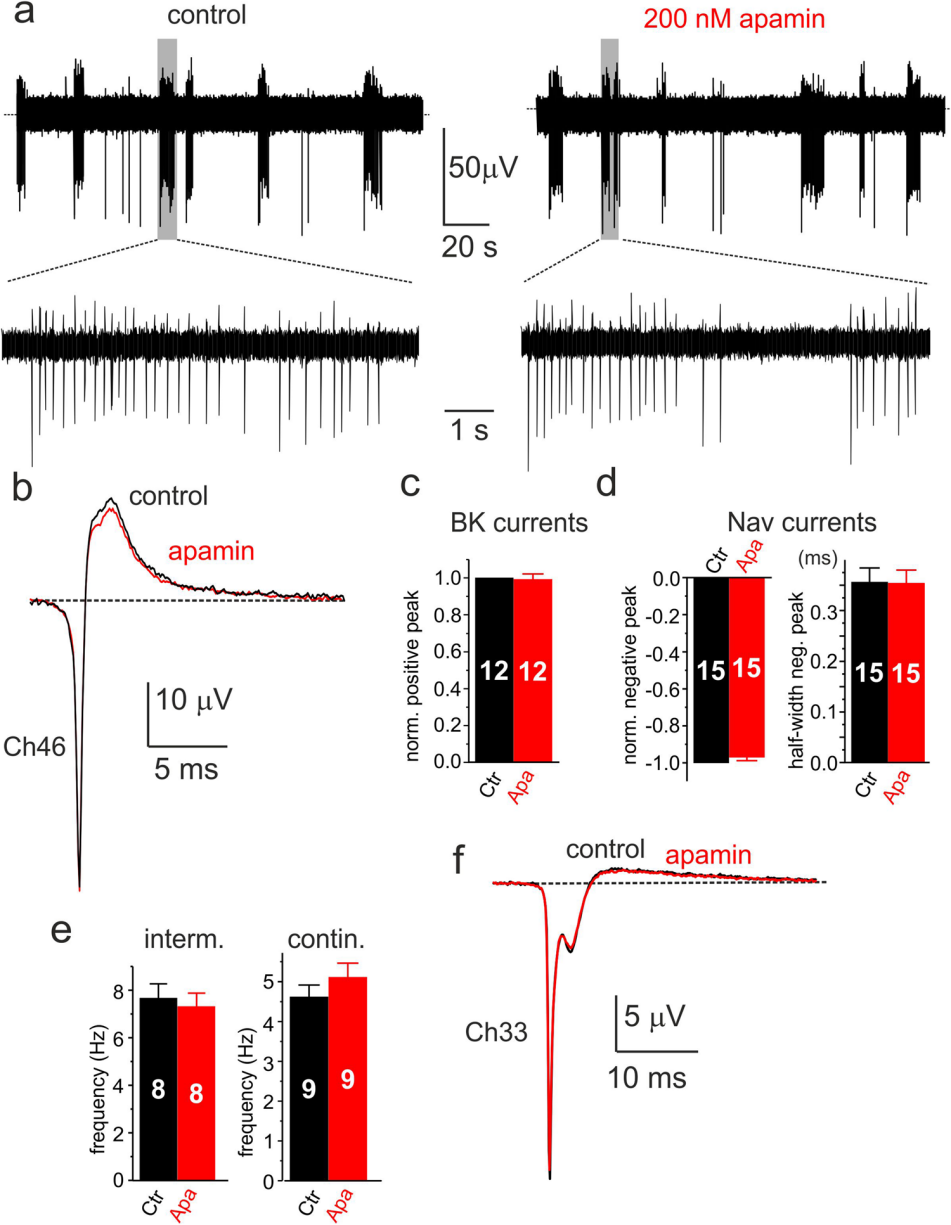
Apamin has no effects on negative-going eAPs. **a** Effects of 200 nM paxilline on the negative-going continuous eAP firings of a rat CC at different time scales. **b** Averages eAPs recorded in control (black; 138 traces) and 200 nM apamin (red; 243 traces). **c** Mean values of the normalized positive peak eAP estimated as indicated in the inset of panel 7b. **d** Mean values of the normalized negative peak and half-width of eAPs. **e** Mean frequencies of intermittent (intraburst) and continuous firing. **f** Average eAPs recorded from a cell with a small positive eAP component. Addition of 200 nM apamin (red trace) produces no changes to the control eAP (black) highlighting the two negative peaks typical of Nav and Cav currents recorded in iAP-clamp. The displayed eAPs are averages of 1053 traces (control; black) and 1559 traces (paxilline; red)

We also tested the effects of the SK channel blocker apamin. Regardless of the concentration (200 nM to 1 μM), apamin had nearly no effects on eAP waveforms and firing frequency (Fig. 9). Figures 9a and b show an example of intermittent eAPs recorded from a rat CC treated with 200 nM apamin. Neither the outward (Fig. 9c) nor the inward eAP amplitude (Fig. 9d) were altered. The inward component, carried by Nav and Cav channels, had unaltered peak and half-width (Fig. 9d), suggesting no effects on Nav and Cav currents that contribute to the negative-going eAPs. Apamin had also no action on the intraburst frequency of intermittent firing and frequency of continuous firing (Fig. 9e). On cells exhibiting eAPs with a slow decaying outward current anticipated by a second negative peak due to the weak expression of BK channels (Fig. 8f), apamin had no action, suggesting that the residual outward current is not carried by SK but rather by Kv and BK channels.

### Nifedipine reduces the inward/outward components of eAPs and helps uncovering the L-type current sustaining the eAPs

L-type voltage-gated Ca^2+^ channels (Cav1) play a key role in generating the spontaneous AP firing of rat [1, 49] and mouse CCs [46, 50]. Cav1 channels contribute to the inward pacemaker current that generates spontaneous iAPs and activates a large fraction of BK channels that controls the falling phase of iAPs. In this way, Cav1 channels regulate CCs excitability and secretion in rat CCs [48, 49].

The coupling between Cav1 and BK channels is evident from the broadening of iAPs during application of the dihydropyridine (DHP) selective Cav1 channel blocker nifedipine (3 μM). In the presence of the DHP, iAPs become larger and broader (see Fig. 4b in [49] and Fig. 4 in [70]), indicative of a robust block of BK channels by the DHP. Block of L-type currents by nifedipine causes also a reduction of iAPs firing frequency in rat and mouse CCs [49, 50], due to the block of Cav currents contributing to cell pacemaking.

The small red trace in Fig. 6a is the time course of Cav currents (Cav1, Cav1.2, Cav3) recorded in AP-clamp conditions when Na^+^ and K^+^ current are blocked by 1 μM TTX and 100 mM TEA. The Cav current exhibits two negative peaks of − 112 ± 12 pA and − 256 ± 26 pA mean amplitude (*n* = 28). Given that Cav1 represents 50% of the total voltage-gated Ca^2+^ channels in rat [12, 49] and mouse CCs [6, 50], we expected a large contribution of L-type channels to the total Ca^2+^ currents. Indeed, addition of 3 μM nifedipine reduced by 82% and 68% of the two Ca^2+^ current peaks sustaining the iAP (not shown).

**Fig. 10:**
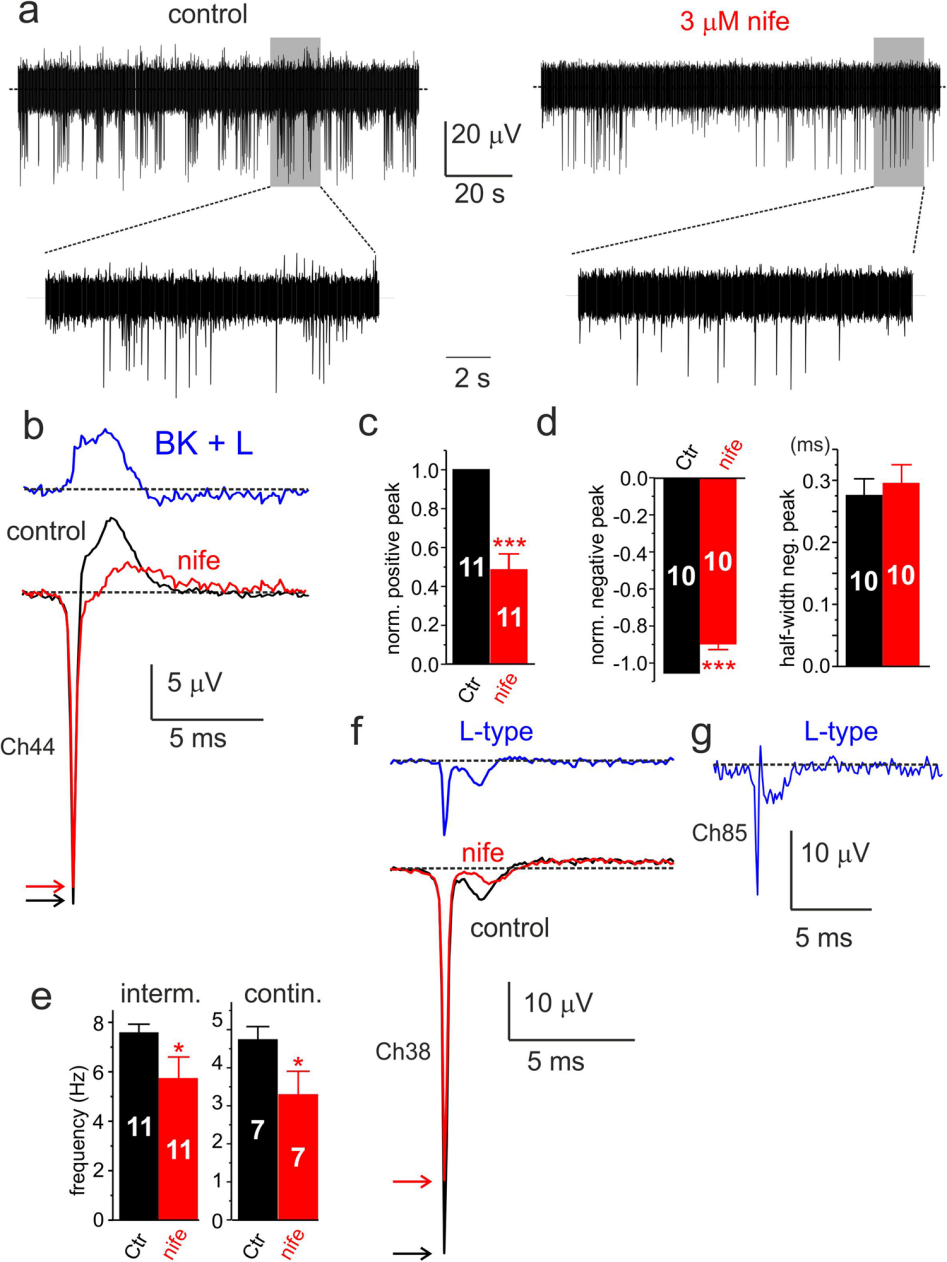
Nifedipine reduces the positive and partially the negative eAP components and in some cell uncovers the contribution of L-type channels to eAPs. **a** Effects of 3 μM nifedipine on the negative-going continuous eAP firings of a rat CC at different time scales. **b** Averages of eAPs recorded in control (black; 225 traces) and 3 μM nifedipine (red; 71 traces). On top, the BK plus the L-type current-associated eAP obtained by subtracting the eAPs of control from 3 μM nifedipine. Colored arrows indicate the peak of the negative eAP component. **c** Mean values of the normalized positive peak (***p < 0.001, paired Student’s *t*-test). **d** Mean values of the normalized negative peak and half-width of eAPs (***p < 0.001, paired Student’s *t*-test). **e** Mean frequencies of intermittent (intraburst) and continuous firing (*p < 0.05, paired Student’s *t*-test). **f** Average eAPs recorded from a cell with nearly no positive eAP component. Addition of 3 μM nifedipine blocks partially the two negative peaks and uncovers the time course of L-type currents blocked by the DHP. The displayed eAPs are averages of 107 (control; black) and 150 traces (nifedipine; red). On top is shown the difference between control and 3 μM nifedipine eAPs that uncovers the L-type calcium current component. **g** Same recordings as in panel **f** but from a different cell with little outward K.^+^ currents. The resulting L-type current (blue trace) is obtained from the subtraction of average eAPs in control (17 traces) and 3 μM nifedipine (38 traces)

We then tested the effects of 3 μM nifedipine on MEA recordings to estimate the changes of eAPs firing frequency and waveforms, and possibly to uncover the time course of L-type currents. Figure 10 shows an example of how 3 μM nifedipine alters the frequency and shape of negative-going eAPs. There is a net decrease of firing frequency and marked effects on the shape of eAP (Fig. 10a,b). Nifedipine blocks by nearly half the amplitude of the peak outward eAP (Fig. 10c), as expected from the block of BK channels coupled to L-type channels [49, 59]. The DHP also decreases by ~ 12% (*n* = 10) the early negative peak associated to Nav and Cav currents, with no changes to the half-width duration of eAPs (Fig. 10d).

The effects of nifedipine on firing frequency were heterogeneous. In 57% of CCs (*n* = 47), nifedipine reduced the firing rate by 47 ± 6% (*p < 0.05), in 23% blocked completely the firing, and in the remaining 20% caused a 15% increase with respect to control. Pooling together the cells that responded with either an increase or a decrease of frequency, we found that the frequency of intermittent and continuous firing decreased by ~ 20% (intermittent) to ~ 30% (continuous) with respect to control (Fig. 10e). Interestingly, in rat CCs that exhibited eAPs with no clear outward components, nifedipine uncovered a nifedipine-sensitive eAP (blue trace in Fig. 10f) that resembles the expected time course of L-type currents (Fig. 6a). The resulting eAP exhibits an early negative peak significantly lower than the negative eAP peak associated with Nav channels. Figure 10g shows a second example of nifedipine-sensitive eAP associated to L-type currents derived from a CC with little BK currents as in panel f.

## Discussion

Our data show that spontaneous eAPs can be easily recorded in rat CCs that adhere on metallic MEAs. The eAPs on MEAs possess the same firing modes of iAPs recorded with glass pipettes in perforated patches, proving unequivocally that spontaneous firing is an intrinsic property of rat CCs. iAPs do not derive from altered cell excitability induced by the patch clamp glass pipette attached to cells in culture [49, 50, 72] or in slices of the adrenal gland [51, 54]. Simple adhesion of CCs to the external microelectrode is sufficient to reveal eAPs with firing modes similar to those recorded in current-clamp conditions (Fig. 3). CCs on MEAs respond also to the modulatory effects of muscarinic agonists (Fig. 4a), suggesting that CCs preserve their physiological function when adhering to metallic MEAs.

Our data show also that regardless of their firing mode (continuous or intermittent), eAPs occur as negative- or positive-going voltage signals. The former resemble the C-type eAP recorded by Schätzthauer and Fromherz [61] in Retzius neurons of the leech using field-effect transistors (FETs) and the latter the ohmic B-type described by [24, 42] in the same neurons. The “negative going” eAP has the same time course of the ionic currents recorded in voltage-clamp using an iAP command as voltage stimulus (Figs. 5 and 6). That is to say, negative-going eAPs reflect the voltage drop at the cell-microelectrode junction generated by the local flow of ionic currents passing through the cell membrane in tight contact with the microelectrode junction during an AP [53, 61]. Thus, MEA recordings of CCs activity provide a direct estimate of the inward/outward currents sustaining spontaneous eAPs over a population of cells.

### Negative-going eAPs represent the Na^+^, Ca^2+^, and K^+^ currents passing through the cell-microelectrode junction during an AP

The eAPs recorded with MEAs display different waveforms depending on the cell-microelectrode coupling and the density of channels expressed at the cell contact region [28, 57, 60, 61]. In the classification of Fromherz et al., eAPs with a positive peak in the rising phase of the AP (A-type) or with a positive peak at the maximum of the AP (B-type) are associated with the capacitive current through the attached membrane or with an enhanced ohmic current, respectively [24, 42, 74]. Alternatively, eAPs with a negative transient during the rising phase and a weaker positive peak in the falling phase of the AP are associated with the voltage drop caused by the ionic currents passing through the cell-microelectrode junction [53, 61] (C-type).

Here, we provide evidence that negative-going eAPs recorded with MEAs on rat CCs reflect the time course of the inward Na^+^/Ca^2+^ and the outward K^+^ currents that sustain spontaneous eAPs. We show that in several rat CCs, some negative-going eAP strongly resembles the time course of Nav currents recorded during an AP-clamp (Fig. 5i). These eAPs derived either by the absence of outward K^+^ currents (Figs. 5e, 9f, and 10f) or by blocking BK and Kv channels with TEA or paxilline (Figs. 7f and 8f). In both cases, the eAPs exhibited the “dual negative peaks” typical of the Nav currents sustaining the iAP (Fig. 5i): a first predominant negative peak associated with the rising phase and a second weaker negative peak associated with the falling phase of the iAP. These CC recordings resemble strongly the eAPs of leech neurons recorded with silicon FETs (see Fig. 2b,c in [61] and of other mollusc neurons in contact with planar metal electrodes [60]).

Further evidence that eAPs provide a genuine measure of the ionic currents flowing through the point-contact junction comes from two other observations. The first regards the block of the negative eAP peak by increasing concentrations of TTX (Fig. 5). The toxin blocks the negative peak of eAPs with the same IC_50_ (~ 10 nM) that blocks pharmacologically isolated Nav currents recorded in voltage-clamp at 22 °C (Fig. 5f–h). In CCs exhibiting large positive peaks (outward K^+^ currents), TTX causes also a partial reduction of the positive peak (Fig. 5d). This is because a reduction of functional Nav channels by TTX reduces the amplitude of spontaneous APs and consequently decreases the number of activated Kv and BK channels that contribute to the positive peak of eAP. The second observation concerns the opposing effects of TEA and paxilline on the negative and positive peaks of eAP. Five mM TEA and 1 μM paxilline block markedly the positive peak (> 95%) and slightly increase the size of the negative peak without altering its half-width (Fig. 7b–d and Fig. 8b–d). The two blockers uncover also the dominant role of BK channels to sustain the positive peak of eAPs. The block of the outward component by paxilline or TEA is either complete (Fig. 8b) or partially complete, preserving a small outward component likely carried by Kv channels (Fig. 7b). This is in line with the experiments on whole-cell clamp in which BK channels carry most of the outward K^+^ currents in rat CCs (80%) and Kv channels contribute to only ~ 20% of the total K^+^ current during an AP command (Fig. 6b). Concerning the activation kinetics, Kv and BK currents display a similar rapid rise but different declines. BK channels decline more slowly than Kv channels and carry significant more current when the iAP is fully hyperpolarized (Fig. 6b). The dominant contribution of BK channels to the outward component of eAPs is also confirmed by the no effects of apamin on eAPs (Figs. 6b and 9b), in agreement with the observation that SK channels do not contribute to the AP upstroke in rat and mouse CCs [72].

### The continuous firing mode is regulated by Na^+^, K^+^, and L-type Ca^2+^ channels

An important finding of our work is that rat CCs plated on MEAs exhibit two spontaneous firing modes (continuous and intermittent) that occur with comparable probability. The continuous one is similar to that reported in rat and mouse cultured CCs [1, 49, 50] or in slices of the adrenal gland [51, 54]. It occurs in 61% of CCs plated on MEAs at 37 °C and exhibits a firing frequency that is 2.4-fold higher than the mean frequency of spontaneous iAPs at 22 °C (4.3 Hz vs. 1.8 Hz; Fig. 2). Notice that a Q_15_ temperature coefficient of 2.4 (from 22° to 37 °C) is in good agreement with the Q_10_ = 2.7 observed for the repetitive firing frequency of squid axon [33] and the Q_10_ = 2.3–2.8 for the rate constants of Nav channel activation of squid axon [45] and node of Ranvier [14]. As clarified by [37] and others [4, 22, 41], an increased rate of activation of Na^+^ and K^+^ channels shorten the AP duration and speed up the ion channel recovery that support the next AP within shorter times allowing the cell to generate higher firing rates.

We also found that the frequency of continuous eAPs on MEAs were sensitive to Nav, Cav, Kv, and BK channel blockers as expected from the effects of these compounds on iAPs. Paxilline and TEA reduced the frequency of continuous eAPs on MEAs (Figs. 7e and 8e) with the same efficacy TEA slowed down the iAPs recorded in current-clamp with a glass pipette (Fig. 6e). Nifedipine also attenuated the frequency in most CCs (Fig. 10e), confirming that L-type channels contribute to the pacemaking current of rodent CCs thanks to the negative voltage-dependent activation, slow inactivation, and high degree of expression of Cav1.3 in CCs [49, 50, 70]. Also low doses of TTX (10 nM) reduced the firing frequency in a fraction of cells. This is in agreement with the idea that reducing the AP amplitude decreases the percentage of Cav, BK, and Kv channels that activate during the AP. The reduced activation of K^+^ channels increases the AP duration while the reduction of Cav channels increases the interspike interval sustaining the firing [71]. On the contrary, apamin has nearly no effect on the spontaneous firing frequency. This is not surprising since apamin is shown to be effective only on slowly firing mouse CCs while has weak effects on fast firing cells [72].

### The intermittent firing mode is more evident on MEAs at 37 °C

An interesting finding of our work is the intermittent eAP firing mode of rat CCs that is highlighted by MEAs. The long lasting bursts of 7.4 s duration interrupted by silent periods of 12.1 s strongly resemble the intermittent firing of iAPs in rat CCs (see [32, 70] and Fig. 3), but it is significantly different from the fast burst firing (0.3–0.5 s duration) repeated every 0.5–1 s observed in mouse CCs [30, 71]. This firing mode in mouse is characterized by short repeated depolarizations of progressively decreased amplitude sustained by a “plateau potential” that increases gradually during the burst [30, 46, 51, 54, 71]. The intermittent firing reported here does not display a clear plateau potential (Fig. 3a) and occurs more frequently on MEAs at 37 °C (39% of cells) than in perforated patches at 22 °C (5% of cells). This suggests two types of considerations. The first concerns the 2.5-fold increase of intraburst frequency (from 3.1 to 7.9 Hz) that is in line with the 2.4-fold increased frequency observed on the continuous firing mode attributed to the increased rate constants of Na^+^ and K^+^ channels activation with temperature. The second regards the reason why the intermittent firing is more frequently observed at higher temperature. A possibility is that temperature changes favor the switch from continuous to intermittent firing, as observed in several neurons with increasing [4, 11, 18, 19, 21, 39, 40, 68] or lowering the temperature [34, 35]. The switch is likely induced by the increased rate constants of Na^+^ and K^+^ channels activation that generate repetitive firing in neurons and whose frequency accelerates with temperature [21, 40]. How this occurs in rat CCs remains to be clarified. Further experiments using ion channel blockers or agonists should be performed to try to convert one firing mode into the other on eAPs and iAPs. In particular, the role of the recently described Na^+^ background current that regulates the firing modes in mouse CCs [54] should be tested, as well as the possible expression of thermosensitive TRP channels [69], whose activation may favor the transition from tonic to burst at higher temperatures. The origin of the intermittent firing pattern on CCs will certainly stimulate future works.

## Conclusions

The aim of this work was to investigate whether CCs possess intrinsic properties suitable to sustain spontaneous firing at rest. We have shown that when plated on metallic MEAs, CCs possess two spontaneous firing modes (continuous and intermittent) that occur with more or less the same probability at 37 °C. The intermittent mode is significantly different from the burst firing described in mouse CCs [30, 71] and is likely responsible for most of the basal release of CAs on rat CCs at rest. A part from this, the most relevant finding of the present work is the discovery that negative-going eAPs uncover the time course of the ionic currents (Na^+^, Ca^2+^, and K^+^) passing through the cell membrane in tight contact with the microelectrode (point-contact region) during spontaneous firing. The early negative component is carried by Nav and Cav currents and displays the characteristic “double inward peak” of the Nav and Cav currents recorded in voltage-clamp under AP commands. The late positive component reflects the outward BK and Kv currents that contribute to the repolarization phase of AP.

In conclusion, taking advantage of the spontaneous firing of cultured rat CCs at basal conditions, we have shown that is possible to identify the ionic currents that sustain AP firing in populations of CCs. From this point of view, rat CCs on MEAs provide an unprecedent user-friendly approach to effectively screen clinically relevant ion channel modulators. Future tests on MEA recordings in slices of the adrenal gland or directly on the entire intact gland [67] will possibly highlight the potential of this technique.

## Availability of supporting data

Supporting data will be fully available on request to the reviewers, editor in chief, and executive editors.
