# Correction to: Two firing modes and well‑resolved Na^+^, K^+^, and Ca^2+^ currents at the cell‑microelectrode junction of spontaneously active rat chromaffin cell on MEAs

**DOI:** 10.1007/s00424-022-02769-6

**Published:** 2022-11-02

**Authors:** Andrea Marcantoni, Giuseppe Chiantia, Giulia Tomagra, Enis Hidisoglu, Claudio Franchino, Valentina Carabelli, Emilio Carbone

**Affiliations:** 1 Department of Drug Science, Laboratory of Cell Physiology and Molecular Neuroscience, N.I.S. Centre, University of Torino, Corso Raffaello 30, 10125 Turin, Italy; 2 Department of Neuroscience, University of Torino, 10125 Turin, Italy

**Correction to** **:** **Pflügers Archiv - European Journal of Physiology (2022)**

**https://doi.org/10.1007/s00424-022-02761-0**

Originally, the article was published with an error. On page 7 in the legend of Figure 3 (remove "on MEAs") and on page 16 (replace "mouse" with "rat").

The original article has been corrected.

